# Atypical presentation of Lynch Syndrome: a case report

**DOI:** 10.1186/1897-4287-9-S1-P10

**Published:** 2011-03-10

**Authors:** Shanna Gustafson, Whitney Ducaine, Dana Zakalik

**Affiliations:** 1 Cancer Genetics Program, Beaumont Hospital, Royal Oak, MI, 48073, USA

## Background

Hereditary Breast and Ovarian Cancer (HBOC) syndrome due to mutations in *BRCA1* and *BRCA2* is associated with an increase in risk for primarily breast cancer and ovarian cancer. Lynch syndrome (LS), due to mutations in *MLH1, MSH2, MSH6,* and *PMS2* is typically associated with an increase in risk for chiefly colorectal cancer and endometrial cancer. Ovarian cancer risk is also increased in LS, however less so than in HBOC.

## Method

We describe a rare presentation of LS, diagnosed in a patient who presented at the age of 41 with a suspected primary ovarian cancer. Her family history was significant for a sister with breast cancer at the age of 29 found to carry a *BRCA1* variant of uncertain significance; a mother who passed away from ovarian cancer; and a father with prostate cancer, and a paternal family history significant for breast cancer, pancreatic cancer, and a possible ovarian cancer. No colorectal or endometrial carcinoma was reported in maternal or paternal family history.

## Result

Calculated BRCAPRO likelihood of a *BRCA1/2* mutation, with a primary ovarian carcinoma as the probands diagnosis, was 90.2% [1]. Full clinical testing for mutations in *BRCA1* and *BRCA2* was pursued and was negative for any deleterious mutations or variants of uncertain significance in this patient.

Pathologic analysis of the patient’s resected ovarian mass reported suspicion of GI or hepatobiliary origin due to positive staining for CK7, CEA and CK20. Due to the history of multiple ovarian tumors in the family; the possibility of a primary GI tumor at a young age in the probands; and the negative HBOC testing; LS genetic testing was pursued and detected a deleterious *MSH2* mutation, IVS5+3A>T. Appropriate testing and surveillance for Lynch Syndrome associated cancers for the patient and at risk relatives can now be pursued.

## Conclusion

This case emphasizes the importance of consideration of Lynch syndrome as well as HBOC in all families presenting with multiple ovarian cancers and the crucial element which pathology plays in accurate risk assessment.
